# Characterization of influenza A viruses with polymorphism in PB2 residues 701 and 702

**DOI:** 10.1038/s41598-017-11625-y

**Published:** 2017-09-12

**Authors:** Alex W. H. Chin, Nathaniel K. C. Leong, John M. Nicholls, Leo L. M. Poon

**Affiliations:** 1 Centre of Influenza Research & School of Public Health, LKS Faculty of Medicine, The University of Hong Kong, Hong Kong, China; 2 Department of Pathology, LKS Faculty of Medicine, The University of Hong Kong, Hong Kong, China

## Abstract

The 701 and 702 positions of influenza PB2 polymerase subunit are previously shown to have roles on host range. Limited polymorphisms at these two residues are identified in natural isolates, thereby limiting the study of their role in the polymerase. In this study, we generated 31 viable viruses by random mutagenesis at this region, indicating that these positions can tolerate a wide range of amino acids. These mutants demonstrated varying polymerase activities and viral replication rates in mammalian and avian cells. Notably, some mutants displayed enhanced polymerase activity, yet their replication kinetics were comparable to the wild-type virus. Surface electrostatic charge predication on the PB2 structural model revealed that the viral polymerase activity in mammalian cells generally increases as this region becomes more positively charged. One of the mutants (701A/702E) showed much reduced pathogenicity in mice while others had a pathogenicity similar to the wild-type level. Distinct tissue tropisms of the PB2-701/702 mutants were observed in infected chicken embryos. Overall, this study demonstrates that the PB2-701/702 region has a high degree of sequence plasticity and sequence changes in this region can alter virus phenotypes *in vitro* and *in vivo*.

## Introduction

The genome of influenza A virus consists of eight viral RNA (vRNA) segments in negative sense. The reassortment of influenza viruses and the high mutational rate of this virus potentially generate novel viruses that favour interspecies transmission. This might facilitate the introduction of newly emerging animal viruses or their reassorants to human populations. Such occasional zoonotic transmissions may cause influenza pandemics^1^. Therefore, it is important to investigate the determinants of the viral genome that might alter the viral pathogenicity and/or tropisms in different host species.

Polymerase basic protein 2 (PB2) is one of the subunits of influenza viral polymerase. It has a cap binding activity and it plays a key role in the “cap-snatching” event during viral RNA transcription^2^. PB2 has been shown to play a critical role in host specificity and adaptations^3^. Avian influenza A viruses mainly consist of a glutamic acid at PB2 residue 627^4^. Previous studies have shown that PB2-E627K mutation of an avian influenza A virus can lead to increased viral growth and transmissibility in mammalian hosts, which contributes to host adaptation to humans^5–7^. Our previous study demonstrated the sequence plasticity of PB2-627 and the discrete phenotypes *in vitro* and *in vivo* ^8^. On the other hand, PB2 residue 701 is another extensively studied genetic determinant for host adaptation. PB2-D701N exhibits elevated virus replication and transcription in mammalian hosts, suggesting a compensatory effect of PB2-701N in the absence of PB2-627K for host adaptation^9–11^. In addition, PB2-D701N mutation promotes both the transmissibility of influenza in guinea pig and the pathogenicity in mice^9, 12, 13^. Sequence analyses have also revealed that PB2 residue 702 shows host specificity, with most avian viruses carrying a lysine and most mammalian viruses carrying an arginine at PB2-702^14^. Previous work further showed that PB2-K702R mutation can enhance polymerase activities^15, 16^.

The amino acids of PB2 in the circulating influenza virus are predominantly aspartic acid or asparagine at residue 701 and lysine or arginine at residue 702^14, 17^. The lack of genetic diversity in PB2-701 and 702 has hindered in-depth understanding of the functions of these host determinants of IAVs. Here, we use a random mutagenesis approach to introduce random mutations at PB2-701 and 702 positions of influenza A virus. This allows us to assess the genetic plasticity of these two residues and determine the effect of different mutations at these residues on viral fitness.

## Results

### Generations of recombinant viruses with random mutations at PB2-701 and 702

To determine the sequence plasticity at the PB2-701/702 region, random mutations of these two residues were introduced to a pHW2000-PB2 plasmid (viral strain used: A/Puerto Rico/8/1934 (PR8)), by site-directed mutagenesis. The mutated plasmid library was then used to generate PR8 mutant viruses by reverse genetics in independent duplicate experiments. Previous studies from us and others have demonstrated that viral particles with a defective polymerase gene can be produced in transfected 293T cells, indicating that the formation of virions is independent of the functionality of vRNP^8, 18, 19^. Progeny viruses were used to infect MDCK cells and 10-day-old embryonated chicken eggs. The viruses collected from MDCK cells and eggs were plaque purified using mammalian MDCK cells and chicken DF-1 cells, respectively. A total of 106 purified viral clones were isolated from both mammalian and chicken cells for further characterization. Sequencing the PB2 genes of these viral clones revealed 31 different replicable viruses with PB2-701 and 702 mutations (Table 1). The amino acids at PB2-701 and 702 showed a great deal of variety, including small, nucleophilic, hydrophobic, amide, acidic and basic amino acids. Aromatic amino acids were also identified at PB2-702. Not surprisingly, the wild-type virus (PB2-701D/702K) was also isolated. Overall, we observed a high genetic plasticity at PB2-701 and 702. Recombinant viruses with amino acids not identified in natural isolates were shown to be viable in both mammalian and avian cell cultures.

**Table 1:** Identification of PB2-701 and 702 mutants isolated in mammalian and avian hosts.

PB2-701/702		
A/A (5.7%)	H/G (0.9%)	S/A (7.5%)
A/E (0.9%)	H/P (4.7%)	S/C 0.9%)
A/K (3.8%)	H/R (5.7%)	S/E (0.9%)
A/S (9.4%)	M/P (4.7%)	S/F (0.9%)
C/G (1.9%)	N/G (5.7%)	S/L (2.8%)
C/T (0.9%)	N/V (0.9%)	S/P (3.8%)
D/G^#^ (1.9%)	N/W (1.9%)	S/R (0.9%)
D/I^ (3.8%)	Q/Q (2.8%)	S/S (1.9%)
D/K* (2.8%)	Q/V (0.9%)	T/P (3.8%)
E/R^¶^ (10.4%)	R/P (3.8%)	V/S (0.9%)
E/A (1.9%)		

Total no. of mutants: 31.

Percentages in brackets indicate the relative frequency of mutants isolated from a total of 106 plaque purified clones.

*Wild type A/Puerto Rico/8/1934.

^^^Same residues 701 and 702 as A/New York/389/2005 2005/01/04, A/ruddy shelduck/Mongolia/686C2/2008(H2N3).

^#^Same residues 701 and 702 as A/duck/Hunan/S11547/2012(H4N9), A/England/558/2003(H3N2), A/TW/229/03(H3N2).

^¶^Same residues 701 and 702 as A/Port Chalmers/1/1973(H3N2), A/England/534/2003(H3N2).

### Effect of PB2-701 and 702 mutations on polymerase activities in mammalian and avian cells

Previous studies have illustrated the effect of PB2 D701N or K702R on polymerase activities in different cell-lines^12, 13, 15^. However, effects of the other PB2-701 and 702 mutations on polymerase activities have not been fully investigated. Here, full-length PB2 segments of 30 mutants and the wild-type PB2 were individually cloned into expression vectors for the luciferase reporter assay. Full-length sequences of these mutated PB2 segments were determined by Sanger sequencing and no additional mutation other than the targeted PB2-701 and 702 mutations was detected. The plasmids expressing wild-type and mutant PB2 were co-transfected with plasmids expressing PB1, PA, NP and a vRNA-like segment with luciferase gene to reconstitute the vRNPs in mammalian 293T cells at 33 °C, 37 °C and 39 °C. A similar reporter assay for avian cells was also used to determine polymerase activities in DF-1 cells as described in the Methods section. The polymerase activities were determined by the luciferase reporter and normalized with the expression of co-transfected green fluorescent protein (GFP) as a transfection efficiency control. In general, the normalized polymerase activity of a vRNP decreases as the temperature increases from 33 °C to 39 °C (Supplementary Fig. S1).

**Figure 1:**
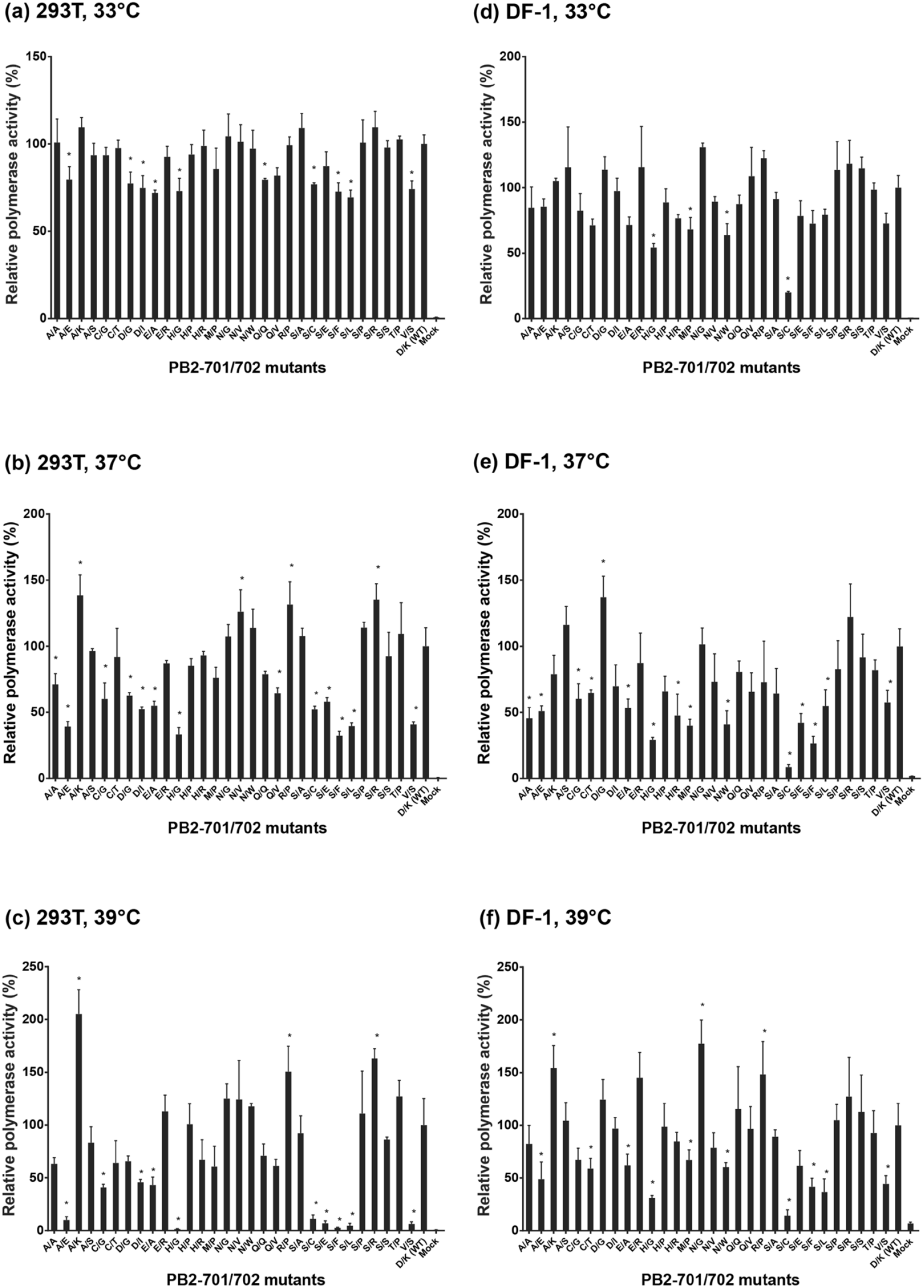
Effect of PB2-701 and 702 mutations on polymerase activity in human and chicken cells. Viral RNP complexes with mutant PB2 genes were reconstituted in human 293T cells at (**a**) 33 °C, (**b**) 37 °C and (**c**) 39 °C, and avian DF-1 cells at (**d**) 33 °C, (**e**) 37 °C and (**f**) 39 °C. Mock represented a negative control without the transfection of PB2 gene. The polymerase activity was determined by the luciferase activity normalized with GFP expression. The relative polymerase activity was expressed in relative to the wild-type activity at the corresponding temperature, which was set as 100%. Data was presented as mean ± 1 s.d. (n = 3). (*p < 0.05 by Student’s *t*-test).

In the mammalian cells at 37 °C, vRNPs carrying PB2-701A/702K, 701N/702V, 701R/702P and 701S/702R had significantly enhanced polymerase activities compared to the wild-type. On the contrary, polymerase activities of vRNPs carrying PB2-701A/702E, 701H/702G, 701S/702F, 701S/702L and 701V/702S were reduced by up to 50% polymerase activity of the wild-type. At 39 °C, effects of these PB2 mutations on polymerase activities in 293T cells were also similar. However, the difference between polymerase activities of the wild-type and the PB2 mutants was more remarkable at this temperature. Reconstituted vRNPs with PB2-701A/702E, 701H/702G, 701S/702E, 701S/702F, 701S/702L and 701V/702S had less than 10% polymerase activity of the wild-type vRNP at 39 °C. At 33 °C, mutants with low polymerase activities were reduced to only around 70–90% of the wild-type activity, i.e. the reduction was not as high as at 37 °C and 39 °C (Fig. 1a–c). Overall, distinct polymerase activities of mutant vRNPs have been observed in mammalian cells. Higher temperature exerted more dramatic influence on polymerase activities of the PB2-701 and 702 mutants.

In DF-1 cells at 37 °C, only PB2-701D/702G led to increased polymerase activity compared to the wild-type. Notably, this mutant led to a slight decline of polymerase activity in 293T cells at the same temperature. At 39 °C, enhanced polymerase activities were observed in vRNPs with PB2-701A/702K, 701N/702G and 701R/702P. Viral RNP with PB2-701A/702E, 701S/702C, 701S/702F and 701H/702G still had substantially reduced polymerase activities (less than 50% of wild-type) in avian DF-1 cells at both 37 °C and 39 °C. Polymerase activities of vRNP mutants in DF-1 at 33 °C showed similar trends as those at 37 °C and 39 °C, but the differences were less remarkable (Fig. 1d–f).

### Correlation between the structural predictions and the polymerase activities in 293T cells at 37 °C

**Figure 2:**
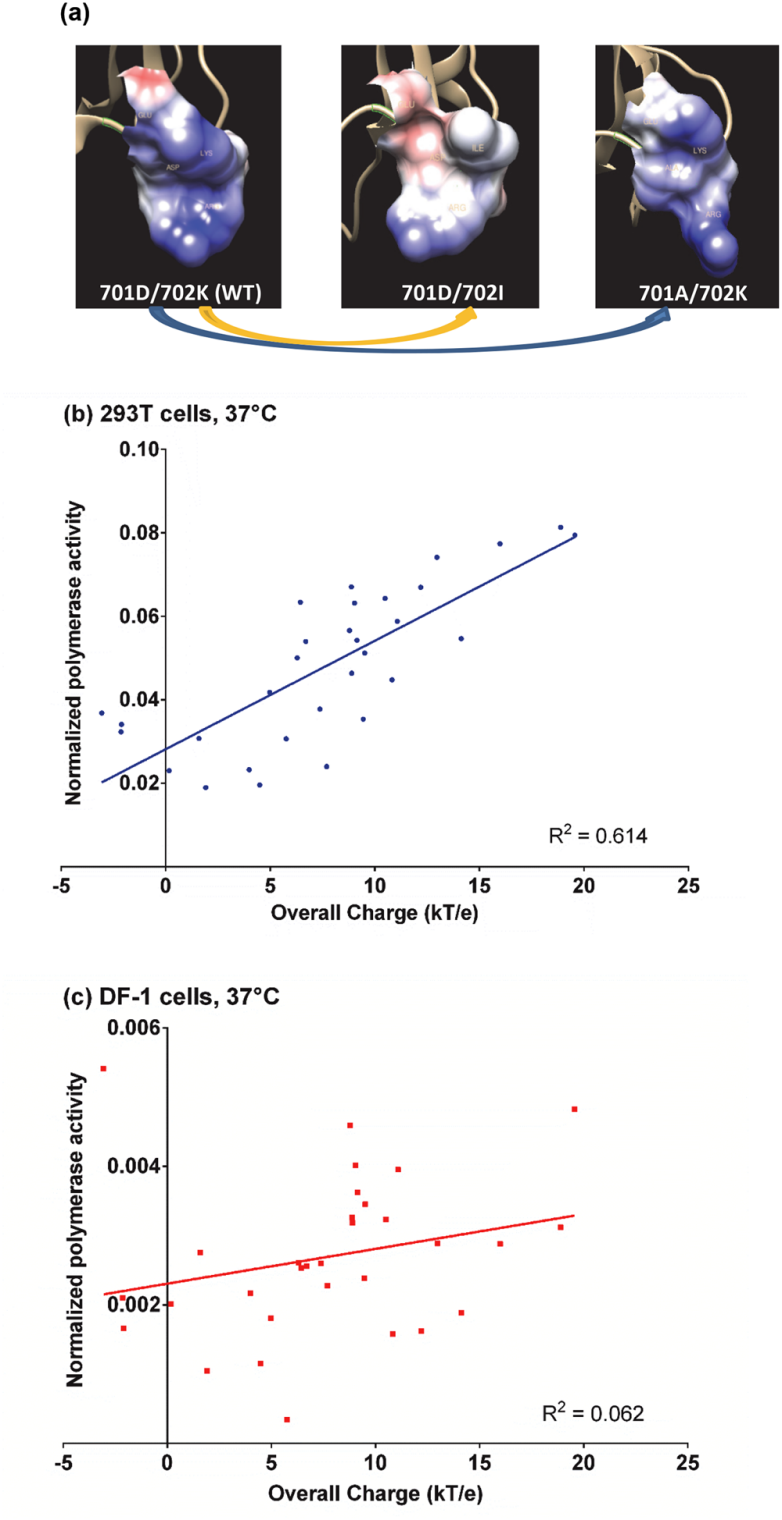
Correlation between surface electrostatic charge and polymerase activity. (**a**) Structural model of the PB2 C-terminal was predicted by the web-server Geno3D and visualized with Chimera. The protein surface was presented from PB2 residues 700 to 703 (left). Coulomb’s law was applied to estimate the electrostatic potential, in which blue and red color indicated positively and negatively charged surface respectively. A change in surface electrostatic charge were observed with PB2-701 or PB2-702 mutation (middle and right). Correlation analyses were performed between overall surface charge of the PB2-700 to 703 region and polymerase activities in (**b**) 293T cells and (**c**) DF-1 cells at 37 °C. Overall surface charge, in the unit of kT/e, was defined as the sum of the charges from residues 700 to 703 determined by Coulomb’s law.

In order to anticipate the effect of PB2-701 and 702 mutations on surface charge distributions, protein models of PB2-701 and 702 mutants (PDB ID: 3CW4, 3L56 and 2GMO) were studied using automatic web server Geno3D. Chimera was used to predict the surface electrostatic charges of the mutants. The surface of the PB2-700 to 703 region showed a differentiated surface charge in different PB2 mutants. The predicted charge distributions of wild-type PB2-700 to 703 region were negative around residue 700 and positive from residues 701 to 703 (Fig. 2a, left). With PB2-K702I mutation, the surface of the region became more negative (Fig. 2a, middle). In contrast, PB2-D701A mutation made the whole surface of the PB2-700 to 703 region positively charged (Fig. 2a, right).

The overall surface charge of PB2-700 to 703 region of different mutants was estimated by Coulomb’s law with Chimera (Supplementary Table S1). The correlation between overall surface charge and polymerase activities of the mutants were determined. A positive correlation was observed between the predicted overall surface charge and polymerase activity in 293T cells at 37 °C (R^2^ = 0.614; Pearson correlation). Mutants leading to a more negatively charged surface of the PB2-700 to 703 region tended to enhance polymerase activity in 293T cells at 37 °C (Fig. 2b and Supplementary Table S1). In contrast, as some of the mutants behaved quite differently in DF-1 and 293T cells (e.g. PB2-701A/702K, 701D/702G, 701N/702V, 701N/702W and 701R/702P; Fig. 1b vs e), the correlation between overall surface charge and polymerase activity was not observed in avian cells (R^2^ = 0.062, Fig. 2c and Supplementary Table S1). Overall, the PB2-701 and 702 mutations may modulate polymerase activity in mammalian cells by altering the overall surface charge.

### Effect of PB2-701 and 702 mutations on growth kinetics of recombinant viruses in mammalian and avian cells

To investigate the effect of PB2-701 and 702 mutations on multi-cycle viral replications, several mutant viruses were selected for the growth kinetics assays with mammalian MDCK and chicken DF-1 cells. The selected viruses included those with enhanced polymerase activities (PB2-701A/702K, 701N/702V and 701S/702R), polymerase activities similar to the wild-type (PB2-701E/702R and 701N/702G) and reduced polymerase activities (PB2-701A/702E, 701D/702G, 701H/702G and 701S/702F). The polymerase gene segments (PB2, PB1 and PA) of these plaque purified viruses were sequenced. It was confirmed that no additional mutation other than the PB2-701 and 702 mutations was present in the polymerase of these viruses. Rescued virus carrying the wild-type sequence (PB2-701D/702K) was used as a control. MDCK and DF-1 cells were infected by the recombinant viruses at an MOI of 0.01 and 0.1, respectively. The infected cells were incubated at 33 °C, 37 °C and 39 °C. Progeny viruses were harvested at 12, 24, 48 and 72 hours post-infection (h.p.i.). The viral titres of the harvested viruses were determined by plaque assays with MDCK cells.

**Figure 3:**
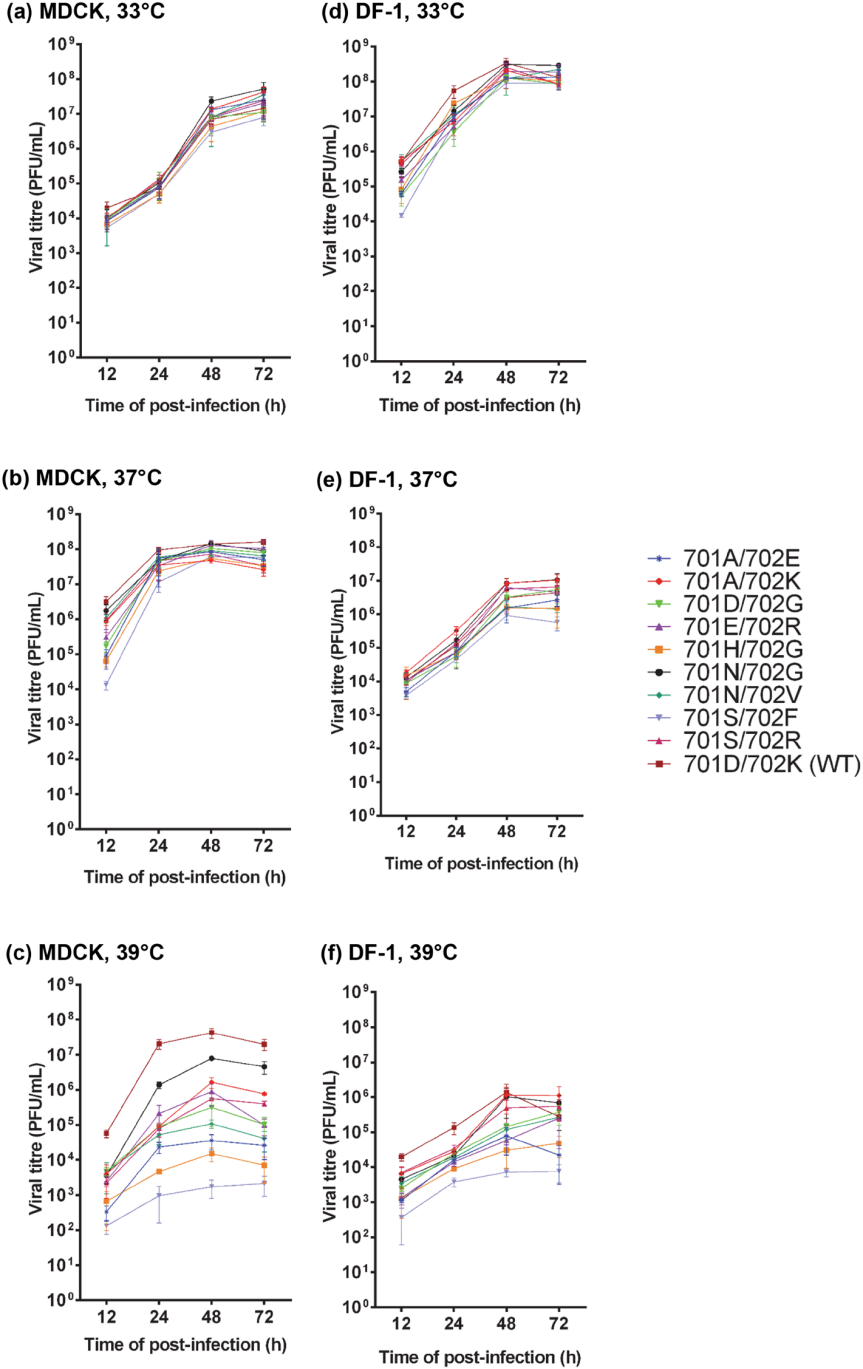
Effect of PB2-701 and 702 mutations on viral growth kinetics in mammalian and avian cells. Mammalian MDCK cells were infected by wild-type and mutant viruses at an MOI of 0.01 at (**a**) 33 °C, (**b**) 37 °C, and (**c**) 39 °C. Avian DF-1 cells were infected by wild-type and mutant viruses at an MOI of 0.1 at (**d**) 33 °C, (**e**) 37 °C, and (**f**) 39 °C. Supernatant was harvested at 12 h, 24 h, 48 h and 72 h post-infection. The viral titre was determined by plaque assay in MDCK cells. Data was presented as mean ± 1 s.d. (n = 3).

In the MDCK cells at 33 °C, all viruses had similar replication rates (Fig. 3a). At 37 °C, viruses carrying PB2-701A/702E, 701D/702G, 701H/702G and 701S/702F had viral titres with more than 10-fold reduction as compared to the wild-type level at 12 h.p.i. All mutant viruses showed similar viral titres as the wild-type virus at 24, 48 and 72 h.p.i. (Fig. 3b). However, at 39 °C, all mutant viruses showed attenuated viral replication as compared to the wild-type. The PB2-701S/702F mutant had more than 1,000-fold reduction in viral titre at 72 h.p.i., compared to the wild-type virus (Fig. 3c). This showed that although some of the mutated vRNPs had more robust polymerase activities, mutant viruses had lower tolerance to higher temperature than the wild-type virus.

In DF-1 cells, all recombinant viruses had similar titres at all time-points at 33 °C and 37 °C (Fig. 3d and e). At 39 °C, the wild-type virus had the most robust viral replication and the viral titre was peaked at 48 h.p.i. In contrast, mutant viruses carrying PB2-701S/702F and 701H/702G had more than a 100-fold reduction in viral titres at 48 h.p.i. as compared to the wild-type virus (Fig. 3f).

Overall, the wild-type virus showed the most robust replication kinetics in both cell types and at different temperatures. The differences between the wild-type and mutant viruses were more remarkable at 39 °C than at 33 °C and 37 °C, showing that the mutant viruses were less tolerant to higher temperature.

### PB2-701A/702E mutant was less virulent in mice

**Figure 4:**
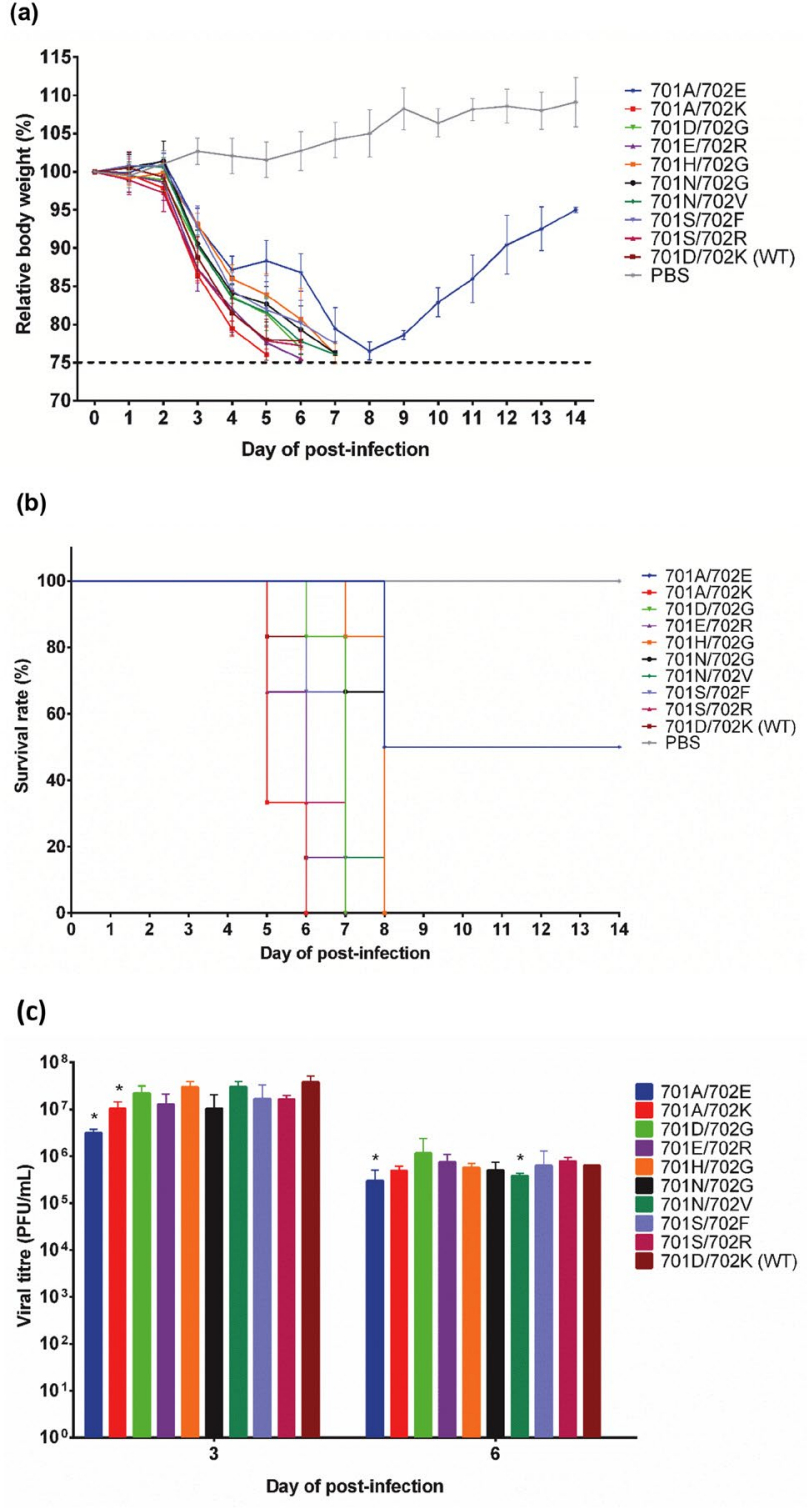
Effect of PB2-701 and 702 mutations on virulence in mice. Female BALB/c mice (n = 6 per group) were intranasally infected by wild-type and PB2-701 and 702 mutant viruses at 1,000 PFU. Mice inoculated with PBS were used as negative control. (**a**) Body weight of the infected mice was measured daily for 14 days. The body weights were presented in relative to the initial body weight (set as 100%). Data was presented as mean ± 1 s.d. Mice with weight loss more than 25% of the initial body weight were humanely euthanized. (**b**) Survival rate of each group was also determined. (**c**) In lung viral titre determination, female BALB/c mice (n = 3 per group) were intranasally infected by wild-type and PB2-701 and 702 mutant viruses at 1,000 PFU as above. Mice were humanely euthanized on day 3 or day 6 post-infection. The lungs were collected and homogenized in ice-cold PBS. The viral titres of the lung homogenates were determined by plaque assay in MDCK cells. Data was presented as mean ± 1 s.d. (n = 3). (*p < 0.05, by Student’s *t*-test).

To examine the virulence of PB2-701 and 702 mutants in mice, selected recombinant viruses were intranasally inoculated into 4- to 6-week-old mice (n = 6) at a dose of 1,000 PFU (~4 MLD_50_ of the wild-type virus). All mice except those infected with the PB2-701A/702E mutant reached the humane endpoint and were euthanized in 5 to 8 days post-infection. Three out of 6 mice infected with the PB2-701A/702E mutant reached the humane endpoint on day 8 post-infection, while the remaining 3 mice in the group recovered from the infection. The mice infected with the PB2-701A/702E mutant also showed less weight loss compared to those infected with the wild-type virus throughout the course of infection (Fig. 4a and b). Viral replication in mice was characterized by determining the viral titre of mouse lungs at day 3 and day 6 after intranasal inoculation (n = 3) with 1,000 PFU of different viruses. The PB2-701A/702E mutant infected mice had a lung viral titre 10-fold lower than the wild-type infected mice at day 3 post-infection (Fig. 4c), although the histochemical staining of the infected lung tissues showed similar pathology in wild-type and mutant viruses infected mice lungs (data not shown). Overall, amongst all studied PB2 mutant viruses, only the PB2-701A/702E mutation could obviously reduce the virulence of the virus in mice in terms of weight loss, mortality and viral replication in lungs.

### Tissue tropism of PB2-701 and 702 mutants in chicken embryos

**Figure 5:**
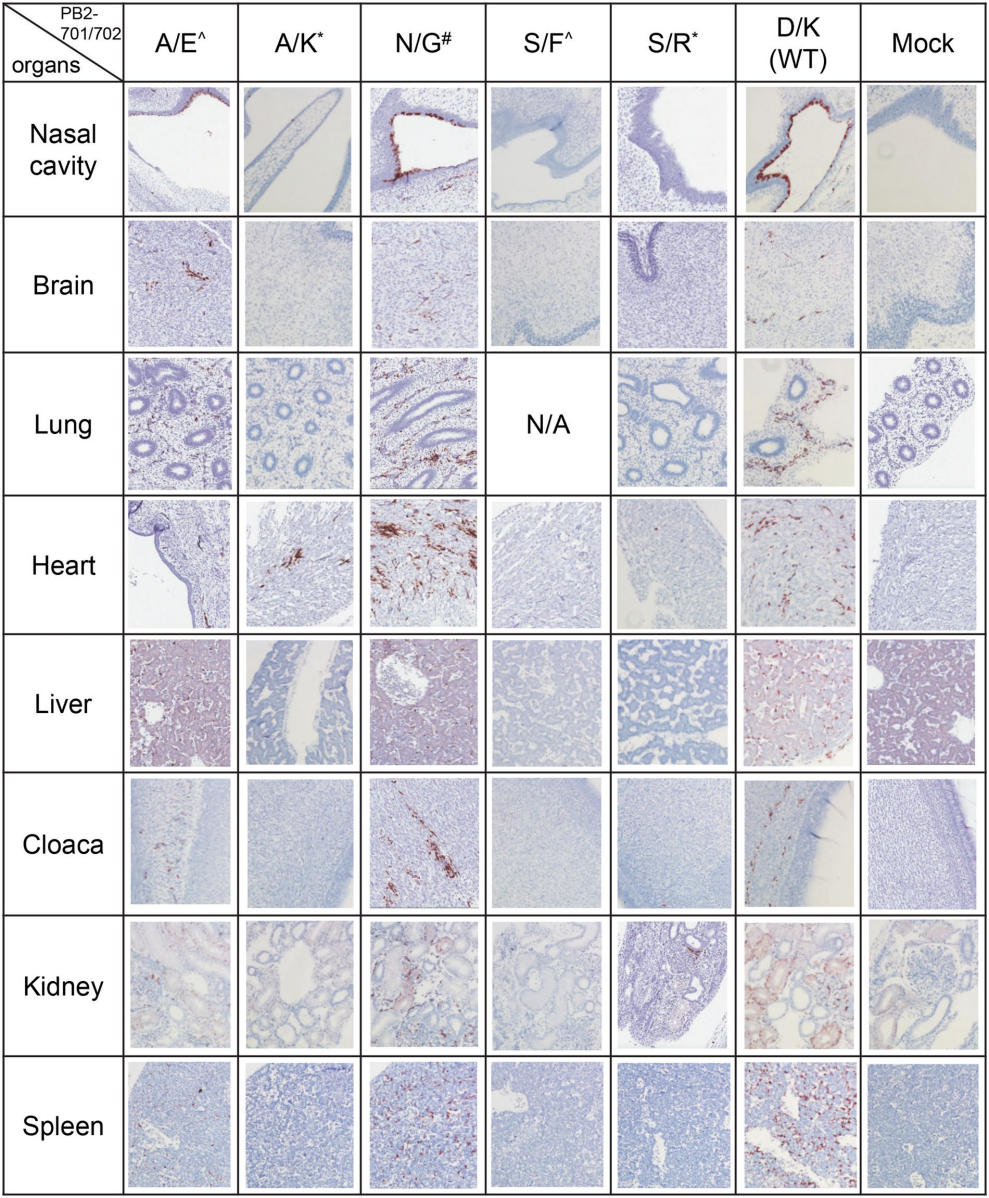
Differential tissue tropism of PB2-701 and 702 mutants in 12-day-old chicken embryos. Embryonic chicken eggs were inoculated with wild-type and PB2-701 and 702 mutant viruses at 100 PFU. PBS was inoculated as mock infection control. The chicken embryos were formalin-fixed at 48 h.p.i. Longitudinal sections of the chicken embryos were H&E stained and immunostained by anti-NP antibody. ^^^Mutants with polymerase activity lower than wild-type. *Mutants with polymerase activity higher than wild-type. ^#^Mutant with polymerase activity similar as wild-type.

Mutations in PB2 have been previously shown to alter tissue tropism in avian host^20^. The prototype virus and its mutants in this study have a lysine at the PB2-627 position, yet these viruses have reasonably robust replications in chicken cell cultures at 37 °C or below. It was of our interest to examine whether the studied PB2-701/702 mutants would have different tissue tropisms in avian hosts. Ten-day-old embryonic chicken eggs were infected by selected mutant viruses in duplicate. Infected chicken embryos were collected and formalin fixed at 48 h.p.i. The viral titres of the allantoic fluid of the eggs infected by different mutant viruses were similar at the time of embryonic fixation (Supplementary Fig. S2), indicating that these viruses can grow efficiently in eggs. Longitudinal sections of the embryos were stained by haematoxylin and eosin (H&E) and anti-NP antibody. The nasal cavity, brain, lungs, heart, liver, cloaca, kidneys and spleen were observed for the expression of NP proteins, as an indicator of viral infection. Interestingly, different mutant viruses demonstrated distinct tissue tropism in chicken embryos. The PB2-701A/702E and PB2-701N/702G mutants caused extensive infection in multiple organs in chicken embryos as the wild-type virus does. In contrast, embryos infected by mutant viruses carrying PB2-701A/702K, 701S/702F and 701S/702R showed none or limited infection in a few organs (Table 2 and Fig. 5).

**Table 2:** Tissue tropism of PB2-701 and 702 mutants in 12-day-old embryonic chicken.

701	702	nasal cavity	brain	lung	heart	liver	cloaca	kidney	spleen
A	E	++	++	++	++	++	N/A	+	N/A
		+	+	++	++	++	++	++	++
A	K	−	−	N.D.	+	−	−	N.D.	−
		−	−	−	−	−	−	−	−
N	G	++	++	++	++	++	++	++	N.D.
		N.D.	++	++	+	++	++	++	++
S	F	−	−	N.D.	−	−	N.D.	−	N.D.
		−	−	N.D.	−	−	−	−	−
S	R	−	−	−	−	−	−	+	−
		−	−	−	−	−	−	+	−
D^#^	K^#^	++	−	++	++	++	++	+	++
		++	++	++	++	++	++	++	++
Mock infection		−	−	−	−	−	−	−	−
		−	−	−	−	−	−	−	−

− No positive immunolabelling detected.

+ One or few foci of immunolabelled tissue per field of view.

++ Extensive immunolabelling per field of view.

N.D. Not determined.

^#^Wild-type.

## Discussion

Previous studies have revealed that there are key residues in PB2 that act as host determinants for interspecies transmission^12, 15, 21^. Apart from the PB2-E627K mutation, which has been extensively studied, PB2-D701N and PB2-K702R were also found to have effects on host specificity and host adaptation of influenza virus^13, 14^. Limited sequence variations at PB2-701 and 702 residues were found in the natural isolates. This limited diversity has restricted our understanding of the role of these residues in host adaptation. Hence, functional analysis of PB2 mutants bearing amino acids other than the prevailing 701D or N and 702K or R was conducted in this study. Theoretically, there are 400 possible combinations of amino acids at these two residues. Random mutagenesis applied in this study allowed us to greatly reduce the effort requiring for generating viable viruses with different amino acids at these two positions. Plaque purification assay was intentionally used for screening mutants that were fit enough for producing visible plaques in infected cells. In two independent experiments, 31 viruses with different amino acid combinations at PB2-701 and 702, including the wild-type virus (701D/702K), were generated in avian and mammalian hosts. Most of these mutants could not be identified in the natural isolates, indicating that 701D/N and 702K/R of PB2 were not exclusively essential to generate viable virus in mammalian or avian hosts.

The effect of PB2-701 and 702 mutations on polymerase activity was investigated using luciferase reporter assays in mammalian and avian cells. It was found that most vRNPs with PB2 mutations showed similar trends of polymerase activity in the two tested cells, except the one with PB2-701D/702G, which showed an enhanced polymerase activity in avian cells but a reduced polymerase activity in mammalian cells. Although the mutants generally showed similar trends of polymerase activity change at different temperatures, the difference between the mutant and wild-type vRNPs tends to be more remarkable at higher temperature. The temperature effect was also observed in the viral replication assay. Several mutant viruses, such as 701S/702F, 701H/702G and 701S/702E, were more restrictive in viral replication at high temperature in both avian and mammalian cells.

We observed that mutant viruses with low replication rates at 37 °C and 39 °C generally have low polymerase activity, indicating that the reduction of polymerase activity is at least one of the factors leading to the decline of the replication rate. However, mutant viruses with elevated polymerase activity did not necessarily increase the progeny viral titres at later time points. This was similar to our previous findings observed from other PB2 mutants^8^. Viral polymerase can affect the efficiency of transcription and replication of the viral genes. However, there are many other viral or host factors that can affect other stages of the viral life cycle including viral entry, membrane fusion and viral genome release, nuclear import of vRNPs, nuclear export of newly synthesised vRNPs, viral packaging and budding. Any one of the factors controlling various stages of the viral life cycle may become the key limiting factor that controls progeny virus formation, even if the virus has robust polymerase activity^22–25^. For example, a host factor cyclin D3 was recently found to interact with the viral matrix protein 2 (M2). This interaction inhibits the M1-M2 interaction, which in turn impairs the virion packaging and attenuates virus production^26^.

Previous studies reported the alteration of surface charge distribution of PB2 because of PB2-E627K and Q591R mutations^27, 28^. In this study, we simulated the change of surface charge of our mutants at the PB2-700 to 703 region. A positive correlation (R^2^ = 0.614) was observed between the overall surface charge in the PB2-700 to 703 region and polymerase activity in 293 T cells at 37 °C (Fig. 2b). However, such correlation was not observed in avian cells (R^2^ = 0.062) (Fig. 2c). These results suggest that the charge of this region is critical for viral polymerase functions in mammalian hosts. The actual mechanism behind this correlation in mammalian host and its implication to host adaptation need to be further elucidated. Nonetheless, this region is located at the nuclear localization signal (NLS) domain of PB2. The NLS domain has different conformations at different status of PB2^12^. The NLS domain can interact with the PB2-627 or PA endonuclease domain^29, 30^. In addition, the NLS domain can also bind to host factors such as importins^31, 32^.

Most of the mutant viruses tested with the mouse model in this study demonstrated similar pathogenicity in mice, except the PB2-701A/702E mutant. It should be noted that that mice infected with mutants with reduced viral RNP activities (i.e. 701A/702E, 701H/702G and 701S/702F) either had less or delayed mortality. Also, it is observed that the PB2-701A/702K mutant is more virulent than the PB2-701A/702E mutant, indicating that the PB2-702E/K polymorphisms could modulate the virus virulence in mice. The observations in the animal study suggest that although the PB2-701 and 702 positions can tolerate a wide range of amino acids, polymorphism at this region still plays some roles in controlling the virus virulence. Further study is required in order to understand the underlying mechanism of how these positions can modulate the pathogenicity of the virus.

Strikingly, for the first time, this PB2 701/702 region is found to have an effect on tissue tropism in an avian host (Table 2). All of the studied mutants had similar viral titres at 48 h.p.i. in eggs (Supplementary Fig. S2). Some mutants (701A/702E and 701N/702G), which are not detected in nature, showed extensive infections in various organs as the wild-type virus does. By contrast, some mutants (701A/702K, 701S/702F and 701S/702R), which are also not detected in nature, showed limited infection in the embryos. The tissue tropisms of the mutants do not correlate to polymerase activities and nor do they correlate to the viral replication rates of the viruses. For examples, both PB2-701N/702G and 701S/702R mutants showed robust polymerase activity and virus replication in DF-1 cells. In addition, results generated from the 701D/702K (WT), 701A/702K and 701A/702E viruses suggested that both PB2-701 and 702 positions have critical roles in controlling tissue tropism in avian host. The underlying mechanism leading to the difference in tissue tropism is yet to be elucidated.

It has been reported that PB2-D701N enhances nuclear import of PB2 via human host factor importin-α1^33^. The usage of different importin-α isoforms also plays a role in tissue tropism and host adaptation of influenza virus^34^. Further investigation on the role of importin-α isoforms on the PB2-701 and 702 mutants may help us to understand the distinct phenotypes of mutant viruses observed in this study and to further elaborate the role of importin-α in host adaptation of influenza viruses.

Overall, this study successfully demonstrates the genetic plasticity of the PB2-701 and 702 positions by generation of recombinant viruses with random mutagenesis. A number of viable mutant viruses generated showed varying polymerase activity and viral growth kinetics in mammalian and avian hosts. These mutants also demonstrated different degrees of pathogenicity in mice. They also showed different tissue tropisms in chicken embryos, with some mutants causing extensive infections in various tissues. This indicates that, apart from controlling the viral tissue tropism in mammalian hosts, this region also has effects on tissue tropism in avian hosts.

## Methods

### Cells and viruses

MDCK and 293T cells were maintained in minimum essential medium with the supplement of 10% fetal bovine serum (FBS). Chicken fibroblast DF-1 cells were maintained in Dulbecco’s modified eagle medium (DMEM) with the supplement of 10% FBS. Specific pathogen free embryonic chicken eggs were used in this study. Influenza A virus (A/Puerto Rico/8/1934) was generated by reverse genetics as described^35^. All the experiments involving infectious influenza A virus were conducted in biosafety level 2 containment.

### Generation of PB2-701 and 702 random mutant virus library

The site-directed random mutations at PB2-701 and 702 were generated by overlap PCR and the mutant PB2 genes were cloned into pHW2000 vector to generate a PB2-701 and 702 random mutant plasmid library^36^. The following degenerate primers were used for random mutagenesis:

5′-GATTCCTCATTCTGGGCAAAGAANNNNNNAGATATGGGCCAGCA-3′ and 5′-ATGCTTAGTGCTGGCCCATATCTNNNNNNTTCTTTGCCCAGAAT-3′.

Recombinant PB2-701 and 702 mutant viruses were generated by reverse genetics^35^. In brief, The PB2 random mutant plasmid library and the 7 plasmids for other gene segments were transfected into 293T cells. At 72 hour post-transfection, the supernatant was harvested and used to infect MDCK cells and 10-day-old embryonic chicken eggs. After 72 hours of incubation at 37 °C, the progeny viruses from MDCK cells and eggs were subjected to plaque purification in MDCK cells and DF-1 cells, respectively. Individual viral clones were isolated and cultured in the corresponding cells. Viral RNA was extracted from the individual viral cultures and the PB2 genes were sequenced to identify the amino acids at PB2-701 and 702 positions by standard Sanger sequencing.

### Luciferase reporter assay

The polymerase activities of PR8 vRNPs carrying PB2-701 and 702 mutations were determined in 293T or DF-1 cells by luciferase reporter assays as described^37^. In brief, pcDNA3 plasmids carrying the PB2 (wide-type or mutants), PB1, PA and NP genes, together with the pPolI-NS-LUCI (carrying the corresponding PolI promotors for mammalian or avian cells) and pMax-GFP plasmids were transfected in mammalian 293T or avian DF-1 cells in triplicate. After 48 hours of incubation at 33 °C, 37 °C and 39 °C, substrate for the luciferase was added (Steady-Glo luciferase assay system, Promega). The luciferase activities were measured by a luminometer. The normalized polymerase activity was determined as the luciferase activity divided by the expression of GFP, which acted as the transfection efficiency control^8^.

### Structural modelling of PB2-701 and 702 mutants

The structural prediction of the protein model was determined by a protein modelling tool, Geno3D, with three PDB templates (PDB ID: 3CW4, 3L56 and 2GMO). The presentation of the protein model was visualized by Chimera. The electrostatic distribution was determined by Coloumb’s Law, which is based on the magnitude of the charge in particular spatial coordinates on the protein surface.

### Viral replication kinetics assay

MDCK and DF-1 cells were infected by the wild-type and mutant viruses at an MOI of 0.01 and 0.1, respectively, in triplicate. After 1 hour of viral adsorption, the inoculum was discarded and the cells were washed gently by 0.9% NaCl solution (pH 2.0) to inactivate any unattached viral particles^38^. The cells were then washed twice with PBS and replenished with infection medium (MEM supplement with 1 μg/mL TPCK trypsin for MDCK cells; DMEM supplement with 0.2% FBS and 1 μg/mL TPCK trypsin for DF-1 cells). The infected cells were incubated at 33 °C, 37 °C and 39 °C. Progeny viruses were harvested at 12 h, 24 h, 48 h and 72 h post-infection. The viral titres of the harvested viruses were determined by plaque assay with MDCK cells.

### *In vivo* pathogenicity study

Four to six-week-old female BALB/c mice were used in this study. To determine the weight loss and survival rate, each group of mice (n = 6) were intranasally inoculated with wild-type and mutant viruses at 1,000 PFU in 25 μl. The body weights were monitored daily for 14 days. Mice with more than 25% body weight loss were humanely euthanized. To determine the lung viral titre in mice, each group of mice (n = 3) were infected with wild-type or mutant viruses at a dose of 1,000 PFU in 25 μl as described above. The mice were humanely euthanized at 3 and 6 days post-infection. The lungs were collected and immediately homogenized in 1 mL ice-cold PBS. The homogenates were centrifuged and the supernatants were kept at −80 °C before the lung viral titres were determined by plaque assay with MDCK cells. All experimental procedures were conducted in accordance with the standards of humane animal care by the criteria outlined in the “Guide for the Care and Use of Laboratory Animals” prepared by the National Research Council, USA (2011) and approved by the Committee on the Use of Live Animals in Teaching and Research, The University of Hong Kong (http://www.lau.hku.hk/content/ethics/ethics.htm).

### Immunohistochemical study in 12-day-old chick embryos

Ten-day-old embryonic eggs were infected by wild-type or mutant viruses at 100 PFU. After 48 hours incubation at 37 °C, the allantoic fluid was collected for determining the viral titre by plaque assay. The embryos were fixed by injecting 10% formalin into the egg followed by immersion of the complete embryo in 10% formalin. The fixed embryo was longitudinally sectioned and stained by haematoxylin and eosin (H&E) and monoclonal antibody against NP (HB65; ATCC) of influenza A virus.

### Statistical analysis

The bar charts and line graphs were generated by Prism 6. The significance of the difference between two groups of data was determined by Student’s *t*-tests. The difference was considered significant with p < 0.05. The correlation between the predicted electrostatic charge and the polymerase activity in 293T and DF-1 cells was calculated by Pearson correlation coefficient.

## Electronic supplementary material

Supplementary figs and table
