# Supplementary material for: Characterization of influenza A viruses with polymorphism in PB2 residues 701 and 702
Source: Sci Rep. 2017 Sep 12;7:11361. doi: 10.1038/s41598-017-11625-y (PMC5595998; doi:10.1038/s41598-017-11625-y)
Supplement: Supplementary file 1 — Supplementary figs and table [file 41598_2017_11625_MOESM1_ESM.pdf]

Supplementary information

Characterization of influenza A viruses with polymorphism in PB2 residues 701 and 702

Alex W.H. Chin[1,+], Nathaniel K.C. Leong[1,+], John M. Nicholls[2], Leo L.M. Poon[1,*]

[1]Centre of Influenza Research & School of Public Health, LKS Faculty of Medicine, The University of Hong Kong

[2]Department of Pathology, LKS Faculty of Medicine, The University of Hong Kong

[+]these authors contribute equally to this work

*corresponding author (llmpoon@hku.hk)

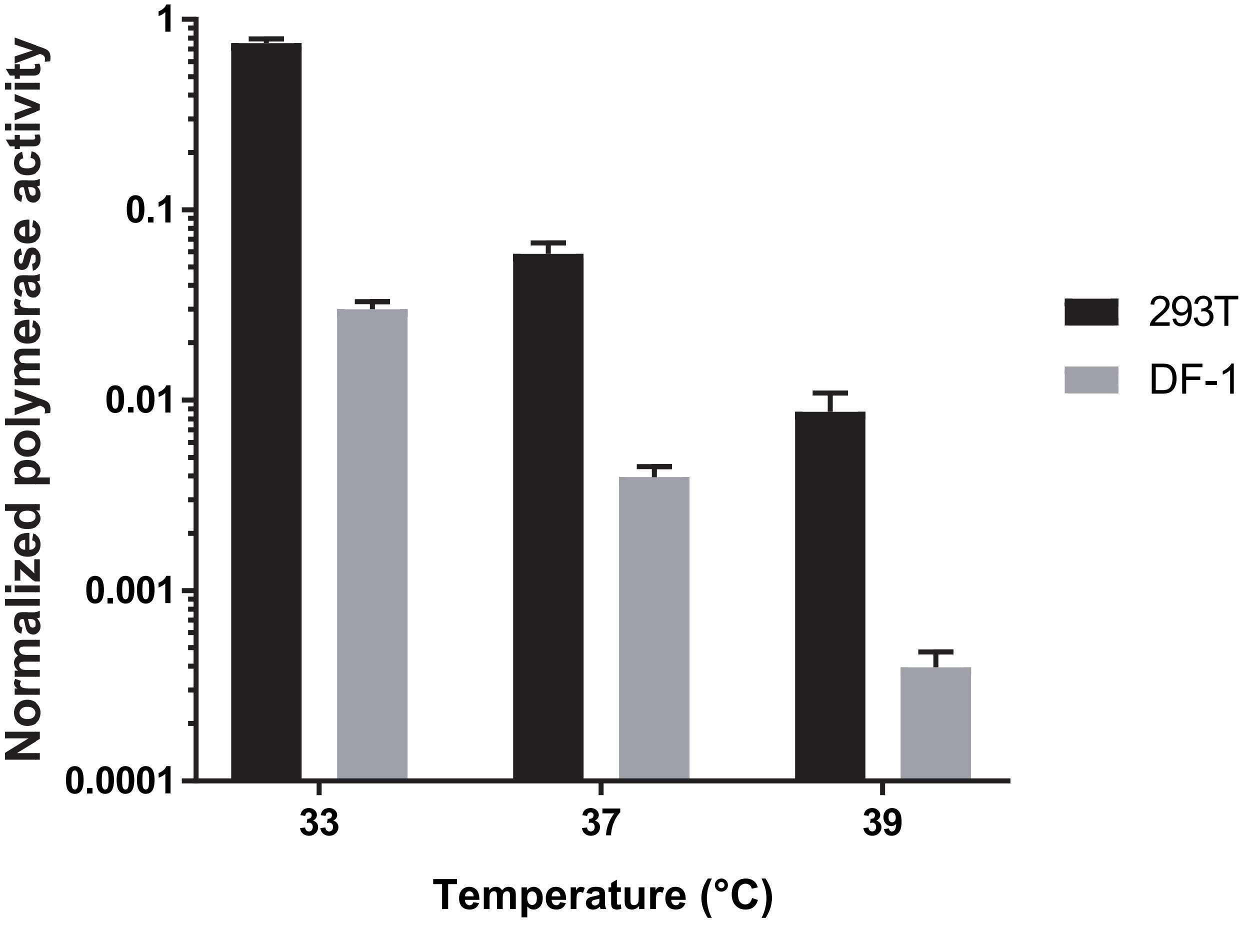

Supplementary Fig. S1 Normalized polymerase activity of wild-type vRNP in 293T and DF-1 cells at different temperatures. Data was presented as mean ± 1 s.d. (n = 3).

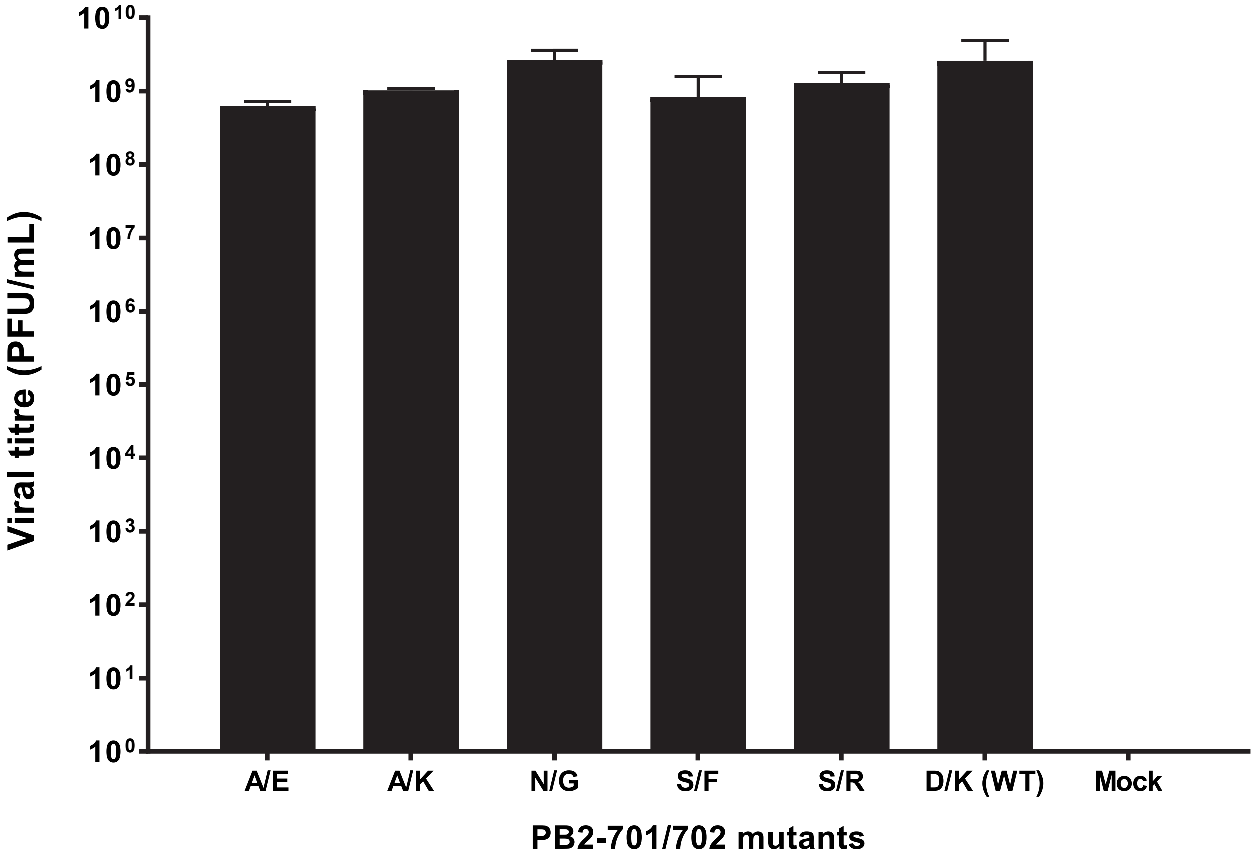

Supplementary Fig. S2 Viral titres of wild-type and PB2-701 and 702 mutants infected embryonic chicken eggs at 48 h.p.i. Data was presented as mean ± 1 s.d. (n = 3).

Supplementary Table S1 Overall surface charge of PB2-700 to 703 region and the polymerase activity of PB2-701/702 mutants in 293T and DF-1 cells

PB2-701/702 mutants	Overall surface charge of PB2-700 to 703 region (kT/e)	293T, 37°C		DF1, 37°C	
		Normalized polymerase activity	Relative polymerase activity to wild-type	Normalized polymerase activity	Relative polymerase activity to wild-type
A/A	4.97	0.0418	71.1%	0.00180	45.6%
A/E	0.16	0.0230	39.2%	0.00201	51.0%
A/K	18.89	0.0814	138.3%	0.00312	78.9%
A/S	8.79	0.0566	96.3%	0.00459	116.2%
C/G	9.45	0.0354	60.1%	0.00238	60.3%
C/T	6.70	0.0540	91.8%	0.00256	64.7%
D/G	-3.06	0.0369	62.7%	0.00542	137.0%
D/I	1.59	0.0308	52.3%	0.00276	69.8%
E/A	-2.14	0.0324	55.0%	0.00210	53.2%
E/R	9.51	0.0512	87.1%	0.00345	87.3%
H/G	4.49	0.0196	33.3%	0.00116	29.2%
H/P	6.29	0.0501	85.2%	0.00261	66.0%
H/R	14.13	0.0547	93.0%	0.00189	47.7%
M/P	10.82	0.0448	76.2%	0.00158	39.9%
N/G	9.03	0.0632	107.4%	0.00401	101.5%
N/V	12.97	0.0742	126.1%	0.00289	73.0%
N/W	12.20	0.0670	113.9%	0.00162	40.9%
Q/Q	8.89	0.0464	78.9%	0.00319	80.7%
Q/V	7.37	0.0378	64.3%	0.00260	65.7%
R/P	15.99	0.0774	131.6%	0.00288	72.8%
S/A	6.44	0.0634	107.8%	0.00254	64.2%
S/C	5.76	0.0307	52.2%	0.00034	8.6%
S/E	-2.11	0.0341	58.0%	0.00166	42.0%
S/F	1.91	0.0190	32.3%	0.00105	26.6%
S/L	3.99	0.0233	39.5%	0.00217	54.8%
S/P	8.88	0.0671	114.1%	0.00326	82.5%
S/R	19.57	0.0795	135.1%	0.00483	122.2%
S/S	9.14	0.0543	92.3%	0.00362	91.6%
T/P	10.49	0.0643	109.3%	0.00324	81.8%
V/S	7.69	0.0240	40.8%	0.00228	57.6%
D/K (WT)	11.08	0.0588	100.0%	0.00395	100.0%
